# The application of deterministic and probabilistic methods of data linkage methods to link population and health data in western Uganda

**DOI:** 10.23889/ijpds.v9i5.2710

**Published:** 2024-09-18

**Authors:** Magdaline Kusuna, Daniel Maina, Herbert Mulindwa, Asinja Kapuru, Caroline Karugu, Daniel Mwanga, Gershim Asiki

**Affiliations:** 1 African Population and Health Research Center; 2 Kagando Hospital

## Introduction

There is increasing interest in record linkage between population-level data and health facility records to support epidemiological research in Africa. However, there is a lack of unique and ubiquitous identification systems for linkage. We applied two common data linkage approaches between population-level data from a health and demographic surveillance system and hospital records in Western Uganda.

## Methodology

We established a data linkage system using deterministic and probabilistic linkage on population level data collected from 23 villages with hospital records. Deterministic linkage entails connecting a pair of records from the clinic and census data using a common identifier. Probabilistic linkage classifies a pair of records from two data sources as belonging to the same individual based on the statistical probability that a common set of identifiers drawn from the two data sources belong to the same individual. The preliminary results are on the deterministic approaches.

## Results

Using deterministic linkage, we fully linked 1061 records from 12/23 villages. Further updates of linkage with characteristics of the linked patients will be summarized in the next two months. Probabilistic linkage data will be presented with the characteristics of participants after the completion of the study, and the agreement between the two methods will be compared.

Note to the results: Presentations during the conference will be done using finalized data.

## Conclusion

Preliminary findings show that the linkage of population and health data is feasible. Findings could be used by researchers to design data linkage studies and answer different types of health and population research questions using innovative data science methods.

